# Parallel shift of DNA methylation and gene expression toward the mean in mouse spleen with aging

**DOI:** 10.18632/aging.204903

**Published:** 2023-07-25

**Authors:** Sangkyun Jeong, Sunwha Cho, Seung Kyoung Yang, Soo A. Oh, Yong-Kook Kang

**Affiliations:** 1 Medical Research Division, Korea Institute of Oriental Medicine (KIOM), Yuseong-gu, Daejeon 34054, South Korea; 2 Genomics Department, Keyomics Co. Ltd., Yuseong-gu, Daejeon 34013, South Korea; 3 Development and Differentiation Research Center, Aging Convergence Research Center (ACRC), Korea Research Institute of Bioscience Biotechnology (KRIBB), Yuseong-gu, Daejeon 34141, South Korea

**Keywords:** methylation shift/drift, hypermethylation, hypomethylation, SiNG-PCR-seq, RRBS

## Abstract

Age-associated DNA-methylation drift (AMD) manifests itself in two ways in mammals: global decrease (hypomethylation) and local increase of DNA methylation (hypermethylation). To comprehend the principle behind this bidirectional AMD, we studied methylation states of spatially clustered CpG dinucleotides in mouse splenic DNA using reduced-representation-bisulfite-sequencing (RRBS). The mean methylation levels of whole CpGs declined with age. Promoter-resident CpGs, generally weakly methylated (<5%) in young mice, became hypermethylated in old mice, whereas CpGs in gene-body and intergenic regions, initially moderately (~33%) and extensively (>80%) methylated, respectively, were hypomethylated in the old. Chromosome-wise analysis of methylation revealed that inter-individual heterogeneities increase with age. The density of nearby CpGs was used to classify individual CpGs, which found hypermethylation in CpG-rich regions and hypomethylation in CpG-poor regions. When genomic regions were grouped by methylation level, high-methylation regions tended to become hypomethylated whereas low-methylation regions tended to become hypermethylated, regardless of genomic structure/function. Data analysis revealed that while methylation level and CpG density were interdependent, methylation level was a better predictor of the AMD pattern representing a shift toward the mean. Further analysis of gene-expression data showed a decrease in the expression of highly-expressed genes and an increase in the expression of lowly-expressed genes with age. This shift towards the mean in gene-expression changes was correlated with that of methylation changes, indicating a potential link between the two age-associated changes. Our findings suggest that age-associated hyper- and hypomethylation events are stochastic and attributed to malfunctioning intrinsic mechanisms for methylation maintenance in low- and high-methylation regions, respectively.

## INTRODUCTION

DNA methylation is a persistent covalent modification that defines the identity of post-mitotic cells for the duration of their existence. Approximately 4% of one billion cytosines in the mammalian genome are methylated [1]. Methylated cytosines are almost exclusively identified in the palindromic CpG dinucleotides, occur in both DNA strands via symmetric cytosine methylation, and are precisely copied from the parent to the daughter strands during DNA replication. This heritability of DNA methylation patterns gives an epigenetic marking of the genome that is stable across multiple cell divisions and hence develops a cellular memory, which makes DNA methylation represent the prototype method of epigenetic inheritance [2]. The methylation status at each CpG site is a flexible substrate for epigenetic modification that can be varied by cellular activity [3, 4]. Changes in DNA methylation have thus been linked to a variety of biological processes including gene regulation and differentiation [5], silencing of transposable elements [6], genomic imprinting [7], and X-chromosome inactivation [8]. DNA methylation patterns normally change dramatically during development, from differentiation of pluripotent stem cells to lineage-restricted stem cells to somatic cells [9]. Once established in somatic cells, methylation patterns are largely fixed and transmitted into daughter cells [1]. In contrast to the biologically regulated and essential DNA methylation changes in early development, the late-life events of DNA methylation change in adult somatic tissue, which gradually occur as environmental components and/or stochastic factors erode the global methylation states [10], do not appear to be programmed or controlled and may be suggestive of age-related pathologies [11].

Epigenetic drift, a process that alters the genomic landscape of DNA methylation as a function of age, impacts the majority of the genome, indicating a widespread dysregulation of DNA methylation patterns with age [12]. This random but continuous event in the genome consequently affects and weakens the highly structured gene regulation frameworks. Thus, DNA methylation can be a molecular culprit behind the age-associated decline in physiological activities and the increasing deterioration of organ and tissue function. Changes in DNA methylation levels with age have been discovered at both the global and local levels. DNA methylation level globally declines with age in various animals and tissues [13–17], which reflects the age-related decrease in DNA methylation of a variety of CpG-rich repetitive sequences [18, 19]. In addition to this global methylation loss (hypomethylation), a methylation gain (hypermethylation) event happens preferentially in CpG-dense gene promoters, especially those of critical developmental genes [20–24]. Many age-hypermethylated genes, including Polycomb group target genes (PCGTs [25]), are also hypermethylated in malignancies and other age-related illnesses [26–28].

We here revisit age-related DNA methylation change using reduced representation bisulfite sequencing (RRBS)-derived methylome of mouse splenic DNA to see if the bidirectional pattern of epigenetic drift appears in spatially clustered (neighboring) CpGs, rather than selective singleton CpG sites, in different categories of genome structure/gene function (promoter, gene-body, and intergenic regions) and if so, what principle underpins the bidirectional methylation change. We further divide the genomic regions either by the density of neighboring CpGs or by methylation level and then examine which one is more closely related to the methylation drift. Finally, to explore the functional repercussion of the bidirectional methylation drift, we investigate the expression levels of 175 epi-driver genes (285 exonic sequences) using the spiking-in a neighbor genome for competitive PCR amplicon sequencing (SiNG-PCRseq [29]) that is specialized for quantification of low-set expression level gene transcripts. Hopefully, our findings will lay the groundwork for future studies studying the significance of epigenetic drift and its effects on cellular physiology as well as establishing a causal role for epigenetic mechanisms in aging. By understanding the epigenetic underpinnings of aging, researchers can begin to uncover the intricate pathways and patterns that are associated with the aging process and develop new interventions to potentially slow the progression of age-related diseases.

## RESULTS

### Age-related decrease in global DNA methylation level in mouse spleen

To investigate age-associated methylation drift (AMD), we performed RRBS (Supplementary Figure 1) on genomic DNA extracted from the spleens of 2-, 6-, 12-, and 23-month-old mice (2m, 6m, 12m, and 23m, respectively; n = 3 per group). We expected a genome-wide methylation change with age in the spleen because it is known that the function and microarchitecture of the spleen gradually deteriorate in older mice, leading to age-dependent impairments in functional immunity [30]. RRBS generated approximately thirty million reads per sample (Supplementary Figure 2A), of which 59% and 23% were uniquely and multiply mapped, respectively, after Bismark alignment (Supplementary Figure 2B). Supplementary Figure 2C depicts the quantitative profile of RRBS-captured *Msp*I fragments to the number of inherent CpG sites in those fragments, highlighting the prevalence of *Msp*I fragments nesting 2-7 CpGs as well as a proportional quantitative representation of the reads aligned to them. A significant proportion (>80%) of individual CpG sites had 10x coverage, which we set to the minimum acceptable depth for methylation ratios (Supplementary Figure 2D).

**Figure 1:**
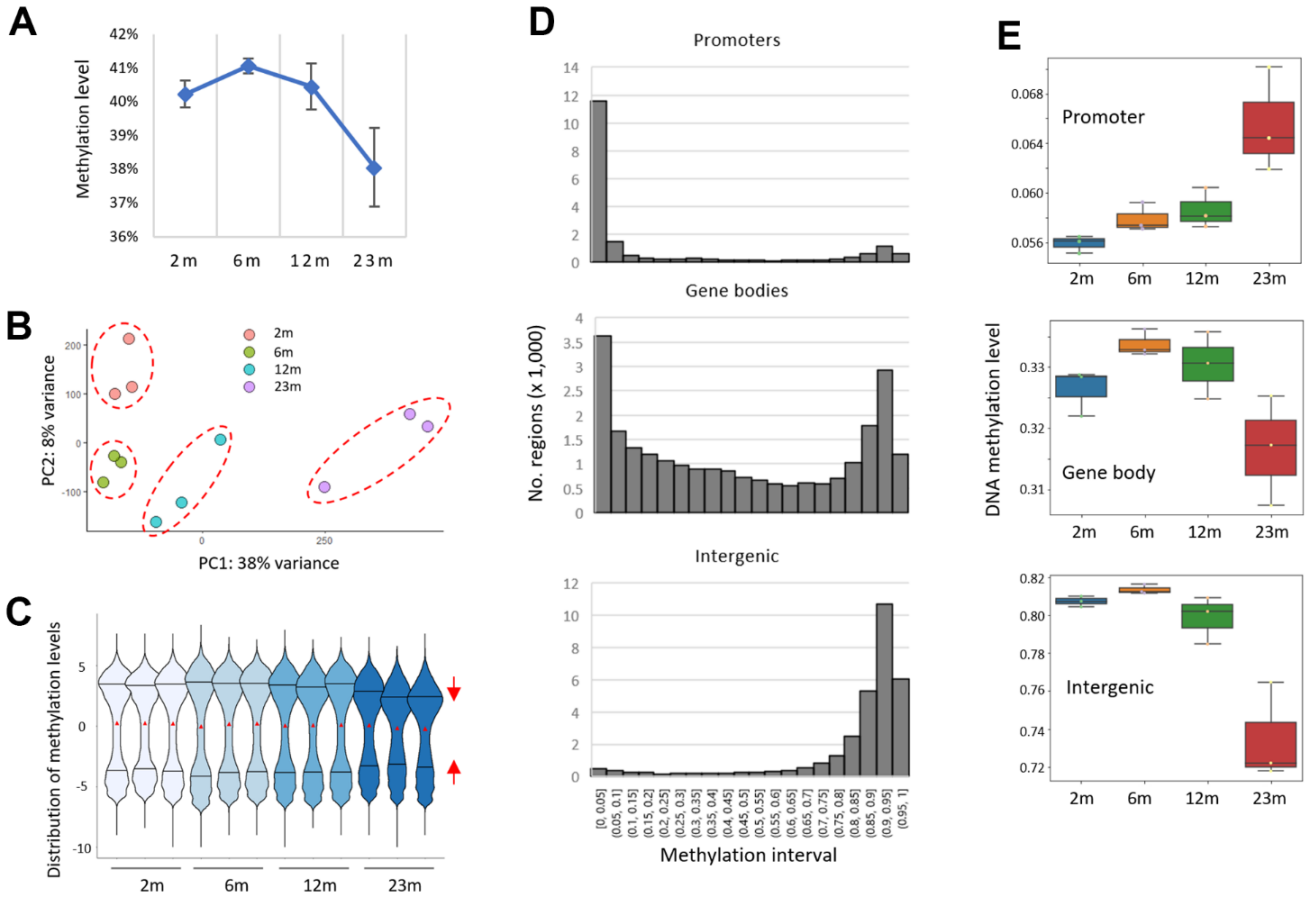
**A bidirectional pattern of DNA methylation drift in the splenic DNA of aged mice.** (**A**) A global decline in CpG methylation level (β-value). RRBS data were obtained from splenic DNA of two-, six-, 12-, and 23-month-old mice (2m, 6m, 12m, and 23m, respectively; n =3 for each). (**B**) Principal component analysis (PCA). A dashed circle group samples of the same age together. (**C**) Distribution of methylation levels (M-value). Red triangles represent the mean methylation levels and the two horizontal lines in the violin plots indicate 25% (lower, Q1) and 75% (upper, Q3) quartile lines. The red triangles reflect the mean methylation levels, whereas the two horizontal lines in the violin plots represent the 25^th^ (Q1) and 75^th^ (Q3) quartiles. The red arrows depict the contraction of the 25% and 75% quartile lines within the 23m age group. (**D**) Histograms showing the distribution of pre-defined regions across successive intervals of methylation levels for different genomic structures/functions (promoters, gene bodies, and intergenic regions). (**E**) Box plots showing the methylation levels across the age groups.

The mean methylation levels of whole CpG sites in samples decreased in older samples (Figure 1A), indicating a decline in global methylation level with age. The intra-group variation of mean methylation level was found to be at its minimum in the 6-month group and increased with age. The decrease was prominent in the 23m age group, which was in agreement with the principal component analysis (PCA) result using M-values (the log2 ratio of the frequencies of methylated CpG versus unmethylated CpG [31]) that revealed the 23m samples far removed from the other younger samples (Figure 1B). Therefore, the results indicated a global, age-associated methylation changes in mice of 23 months of age.

### Region-based methylation analysis proves bidirectional AMD pattern

Violin plot using the M-values revealed that while the distribution of methylation ratios and their mean value were largely even and similar among the samples, the interquartile range (IQR (%) = Q3 – Q1), which is a measure of statistical dispersion of data, was shrunk in the 23m samples (Figure 1C and see also Supplementary Figure 3A for their normal distribution), indicating that the spread of the M-values is reduced in older samples. The result suggested the presence of AMDs with competing modes of action operating on different CpG clusters, which prompted us to conduct a “region”-based study for AMD as opposed to a singleton CpG-based approach. The term ‘region’ here encompasses spatially grouped CpG sites within a defined area that share a structural chromatin setting; henceforth, when we mention a region’s methylation level, we refer to the mean methylation of all the CpGs present in that region. We did this by assigning RRBS CpGs to one of three regional categories with respect to genome structure/function: gene promoters, gene bodies, and intergenic regions. Every CpG that was associated with a gene promoter (transcription start site (TSS) ± 1 kb in length) or gene body (encompassing TSS to transcription end site (TES) minus promoter region) was assigned to the gene, and the mean methylation level of the gene-associated CpGs was obtained collectively. The “intergenic block/region” is defined in the Method section. The frequency of regions with varying CpG counts in the three genome categories is depicted in Supplementary Figure 3B. As shown in Figure 1D, the majority of promoters (74% of 15,292 regions with 5 CpGs or more) were weakly methylated (β-value, the proportion of methylated CpG at a given locus, <5%) in 2m samples, whereas intergenic regions (82.4% of 57,679) were highly methylated (>75%) and gene-body regions (n = 16,554 genes with CpG >0.5 ea per kb) showed a mixed, bimodal methylation state. When the mean methylation levels were compared across ages, the promoters showed hypermethylation as age advanced, whereas the gene bodies and intergenic regions showed hypomethylation (Figure 1E). The extent of AMD was noticeably high from 12 months to 23 months. So, our region-based analysis proved a bidirectional AMD pattern in the mouse spleen. The result suggests that the AMD may cluster spatially as seen in cancer [32], refuting the claim that AMD implicates a larger proportion of singleton CpGs that exhibit solitary DNA methylation changes and are hence less likely to have functional significance.

### Inter-individual heterogeneities in methylation level increase with age

**Figure 2:**
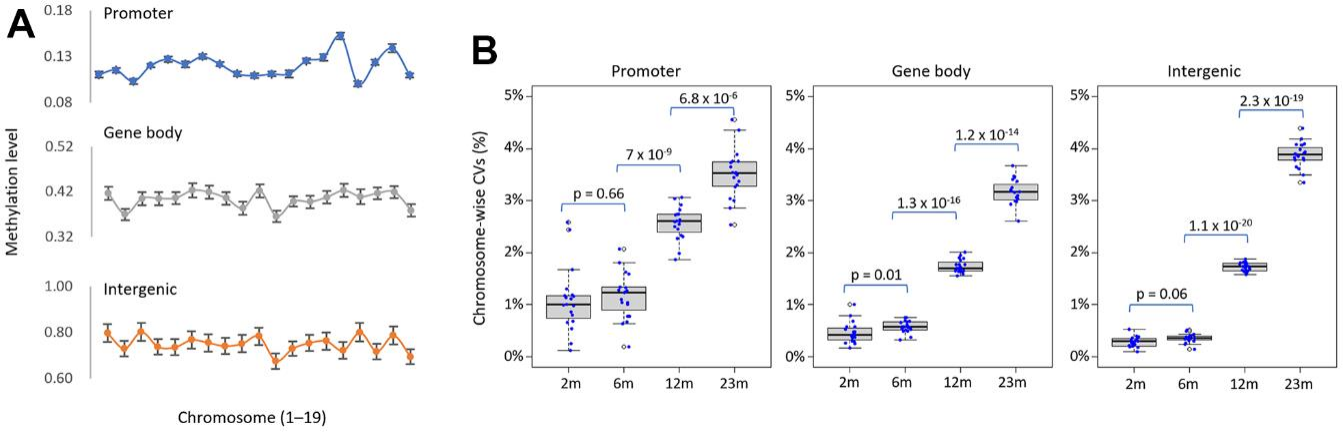
**Increase in inter-individual methylation variations in older spleens.** (**A**) Chromosomal variation in methylation levels of genomic categories. The mean methylation level of each genomic category was determined for each chromosome and shown in the line chart. Error bars represent standard deviation. (**B**) Age-related increase in inter-individual methylation heterogeneity. It shows the distribution of the variance coefficient (CV) of methylation levels across 19 chromosomes (excluding sex chromosomes) for different genomic structures/functions within the same age group. The points on the box plot represent the CV values of each chromosome. The p-values between successive age pairs are shown and were calculated using a paired-sample t-test.

The mean levels of DNA methylation across different genomic categories, including promoters, gene bodies, and intergenic regions, were found to be variable across chromosomes in this study (Figure 2A). Importantly, the standard deviations of methylation levels between individuals appeared to be relatively similar across chromosomes for each genomic category. We calculated the coefficient of variation (CV, the ratio of the standard deviation to the mean) of methylation levels across each chromosome among samples from the same age group to assess the change of inter-individual variability upon aging. The chromosome-wise CVs were persistently low on average in younger samples but significantly higher in older samples, suggesting a rise in heterogeneity with age (Figure 2B). It has been proposed that epigenetic drift, which leads to inter-individual epigenetic divergence, is determined by a lifelong accumulation of epimutations generated by environmental effects and/or intrinsic stochastic variables [33]. Our results with laboratory mice favor stochastic variables for AMD rather than environmental factors; clustered CpGs displaying epigenetic drift may undergo stochastic processes with aging, most likely due to the vulnerability of the region’s chromatin architecture to change.

### AMD pattern at a specific CpG site is related to the density of peripheral CpGs

**Figure 3:**
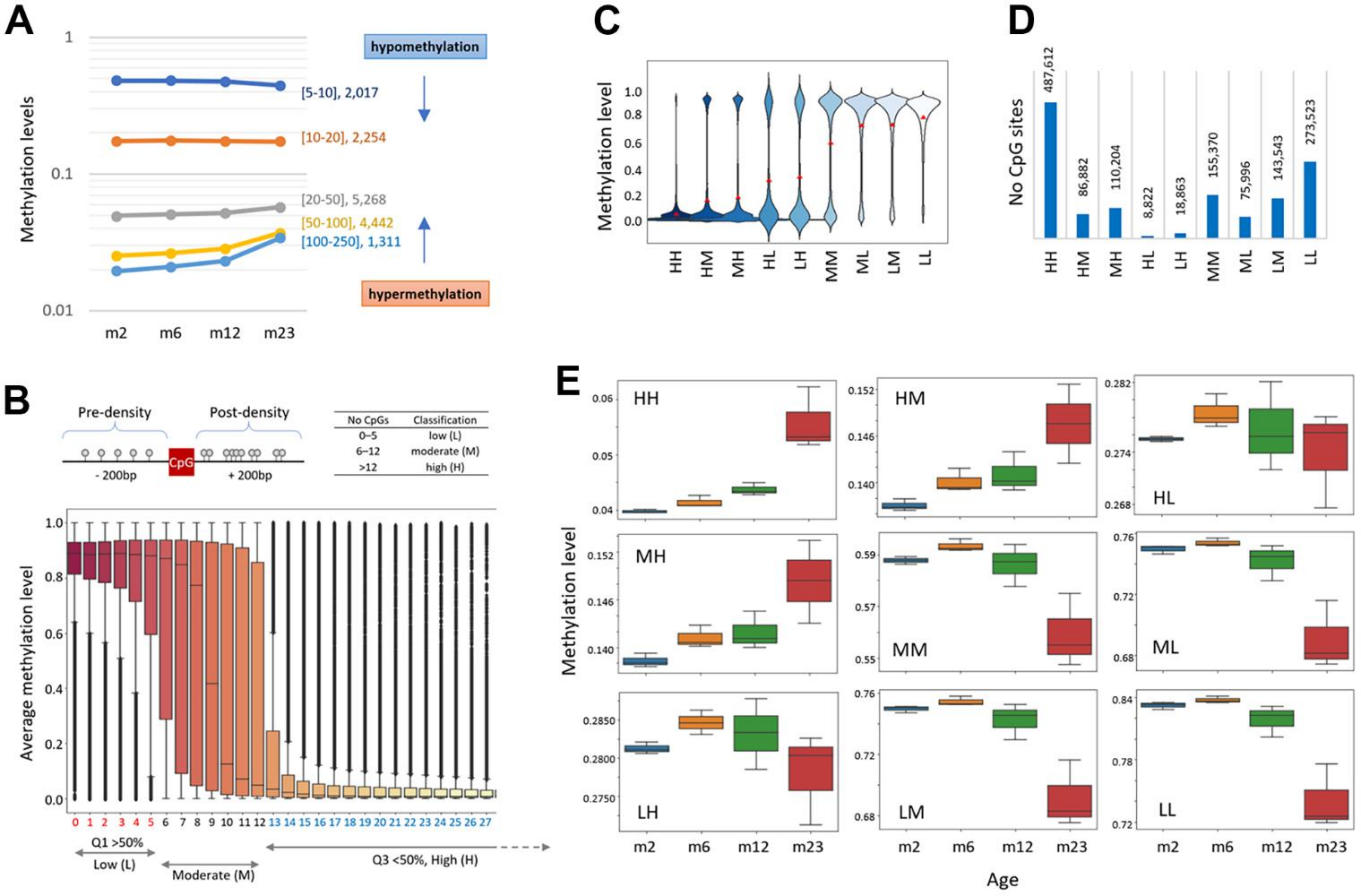
**Age-related methylation-change pattern is linked to the CpG density of the region.** (**A**) Different types of methylation change in areas with varying CpG densities. Promoter regions were divided into five groups based on the CpG density: 5≤CpG<10, 10≤CpG<20, 20≤CpG<50, 50≤CpG<10, or 100≤CpG<250. The number of corresponding promoter regions is denoted by each density category. The arrows represent the CpG-density-dependent increase (hypermethylation) or decrease of methylation (hypomethylation). (**B**) CpG density-based classification of chromosomal CpG sites as low (L, 0–5 CpGs; Q1 >50% methylation), high (H, >12; Q3 <50%), and moderate (M, 6-12; the remainder), respectively. CpG density was determined by counting the number of CpG dinucleotides within 200 bp of a single CpG (top). Box plots show the distribution of methylation levels for CpG sites with identical CpG density. (**C**) Distribution of methylation levels of CpGs in the CpG-density classification groups. Each CpG site is classified as one of the following: HH, HM, HL, MH, MM, ML, LH, LN, or LL. The red dots indicate the average methylations. (**D**) The number of CpG sites assigned to each classification. (**E**) Different patterns of age-linked methylation changes at differently classified CpG sites.

When promoters were classified by CpG density, those with thin CpGs (5-10 CpGs ea per 2 kb, n = 2,017) were relatively highly methylated (48.0%) at two months and became hypomethylated with age, whereas those with thick CpGs (>100 CpGs, n = 1,311) were lowly methylated (1.9%) and then hypermethylated with age (Figure 3A). It suggests that the AMD pattern is determined by the CpG density of the region. We sought to see if there were different types of AMDs in different CpG-density settings, regardless of genomic structure/function. To determine the CpG densities of adjacent sequences, we counted the number of CpGs present in a 200-bp region on either side of a single CpG (Figure 3B, top). The DNA methylation levels at the same density CpG sites were box-plotted, which revealed three qualitatively distinct groups, one with Q1 quartile values larger than 50%, another with Q3 values less than 50%, and the others otherwise (Figure 3B, bottom). Based on this methylation level-based criterion, the density of each CpG site was classified as low (L, 0–5 CpGs), high (H, >12), or moderate (M, 6-12), and each CpG could be assigned to one of nine classifications with two capitals: HH, HM, HL, MH, MM, ML, LH, LN, and LL. The H-labeled CpGs–HH, HM/MH, and HL/LH–were generally undermethylated whereas the L-labeled CpGs–LL and LM/ML–were heavily methylated (Figure 3C). It is commonly considered that HH-CpGs are primarily found in CpG islands (CGIs) and are typically weakly methylated, whereas LL-CpGs are located outside of CGIs (or in ‘open-ocean,’ as the CGI-related description suggests) and are comparatively highly methylated. The HH and HL groups had the most and the fewest CpGs, respectively, of the 1.4 x 10^6^ CpGs available in our RRBS data (n = 487,612 and 8,822; Figure 3D).

We noticed that CpG groups with different density labels exhibit different AMD patterns (Figure 3E). DNA methylation increased with age for the HH and HM/MH CpGs, but declined for the L-/M-labeled CpGs. In the classifications exhibiting a hypomethylation-type AMD, 2m samples were slightly but consistently less methylated than 6m samples, which may indicate that global DNA methylation patterns in mice are still being established at two months of age [34]. Interestingly, the HL/LH group differed from the other H-labeled groups such as MH/HM group and resembled the non-H-labeled (L/M) group. We guess that the methylation level difference at two months between the HM/MH and HL/LH groups (13.7% vs. 27.7%, respectively) could explain the different AMD patterns. Scatter plots of 6m, 12m, and 23m versus 2m samples demonstrated that while the HH and LL classifications were equivalent until 12 months of age, they split into two distinct subsets at 23 months of age, hypermethylation (HH-CpGs) and hypomethylation groups (LL-CpGs; Supplementary Figure 4).

### AMD pattern is determined by the region’s methylation level

**Figure 4:**
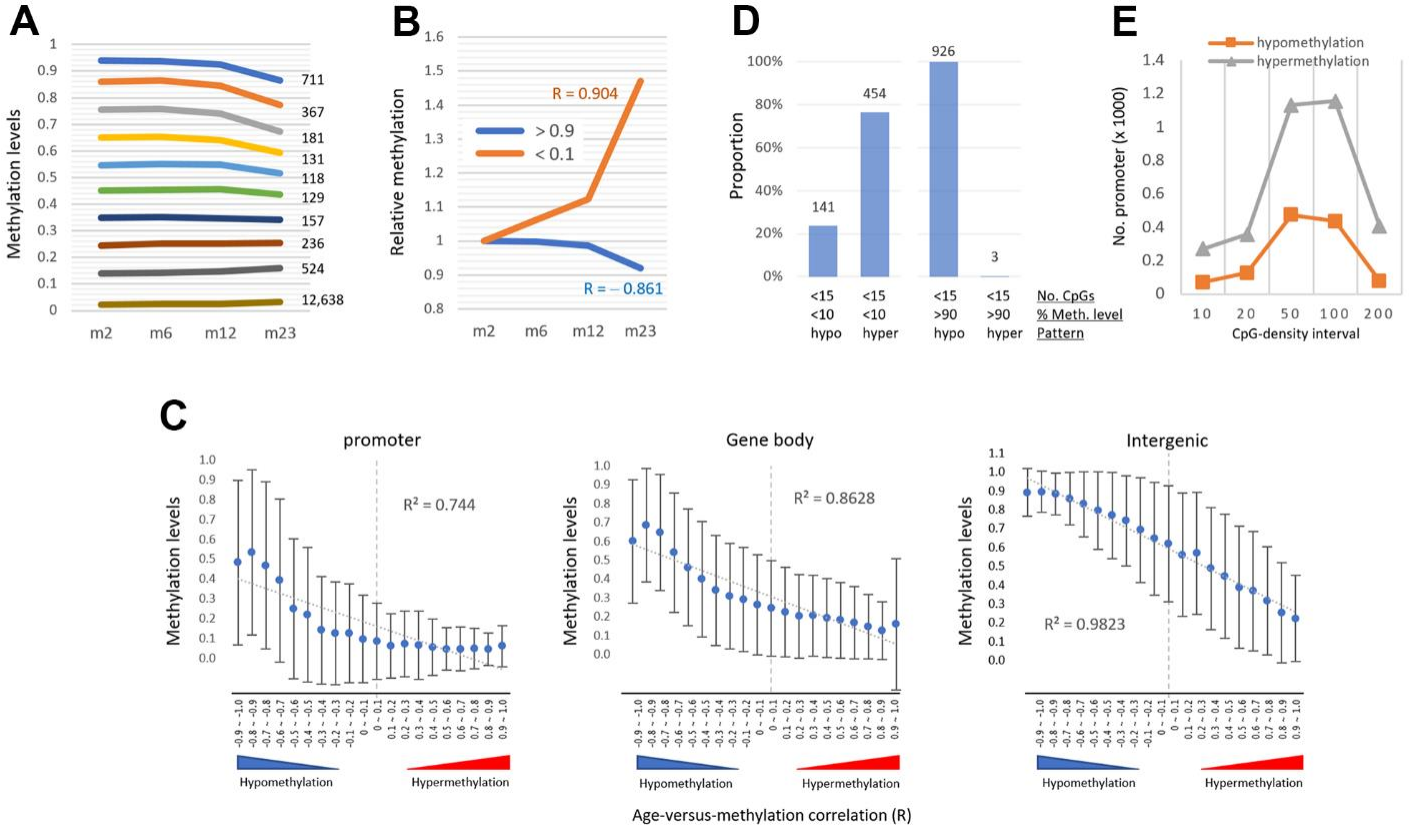
**The pattern of age-associated methylation drift is determined by regional methylation level.** (**A**) Relationship of age-linked methylation change pattern with regional methylation level. Promoters were categorized based on their methylation levels (0.1 intervals of β-values, y-axis) as indicated in different colored lines with their counts. (**B**) Changes in relative methylation levels in promoters with low methylation (<10%, orange) and high methylation levels (>90%, blue) as a function of age. R values indicate Pearson correlation. (**C**) The inverse relationship between the age-versus-methylation correlations (AMR) and the methylation levels of the regions. AMR is a Pearson correlation (R) between regional methylation levels and sample ages, with values approaching +1 and -1 indicating hyper- and hypomethylation, respectively. With the generated AMRs, the areas were grouped (0.1 intervals, totaling 20 intervals ranging from -1.0 to +1.0) and their mean methylation levels (± standard deviation) at two months of age were measured. The R^2^ value on the plot is the determination coefficient between methylation levels and AMRs. (**D**) Prevalence of hypermethylation in promoters with two conflicting variables for age-associated methylation change: low CpG density (<15 ea) favoring hypomethylation, and low methylation level (<10%) favoring hypermethylation (left). Hypomethylation predominates at promoters with both low CpG density and heavy methylation (>90%, right). The regions are counted on the bar. (**E**) The prevalence of hypermethylation in low-methylation promoters (<10%, n = 13,076), regardless of CpG density.

While the bulk of promoters were undermethylated, a minor percentage was considerably methylated. We wondered if these highly methylated promoters experienced the same hypermethylation with age as the lowly methylated promoters. We classified the promoters based on their methylation level (0.1 β-value intervals). Ten % (n = 1,508) of promoters were quite substantially methylated (>50%) and showed a hypomethylation rather than hypermethylation with age (Figure 4A). A similar result was observed from the analysis of gene-body regions (Supplementary Figure 5). The result indicates that when certain regions are initially substantially methylated, they tend to be hypomethylated regardless of the genome structure/function (Figure 4B), indicating that the AMD pattern is methylation level dependent.

The regions in each genomic structure/function were grouped based on the correlation of their methylation levels with sample ages (R; 0.1 intervals each, a total of 20 intervals ranging from -1.0 to +1.0). As shown in Figure 4C, there was a negative relationship between the groups’ R-values and their mean methylation levels at 2 months; as the groups had higher methylation levels, their R-values were more negative (or hypomethylated), whereas as the groups had lower methylation levels, they were more positive (or hypermethylated). It indicates that the pattern of AMD is more closely related to the region’s methylation level. This was most noticeable in the intergenic group (coefficient of determination, R^2^, = 0.982). The promoter category groups with positive R-values showed an exceptionally poor correlation (R^2^ = 0.087) with the methylation levels, probably due to their overall low-lying methylation levels with a marginal difference (~5% difference in the intervals of 0.3 to 1.0) between the intervals.

We then looked into promoters that had an unusual pairing of CpG density and methylation level, such as the one with low CpG density and low methylation level and the one with high CpG density and high methylation level. The incidence of hypermethylation was three times that of hypomethylation (454 vs. 141 promoters; |R-value| >0.8; Figure 4D) for promoters with a low CpG density (15>CpG>4) and a low methylation level (<10%). There were only a few promoters (7 of 1258 promoters) with a high CpG density (>100 CpGs) and high methylation level (>20%), insufficient for a reciprocal analysis. On the other hand, for promoters with a low methylation level (<10%, n = 13,076), hypermethylation prevailed over hypomethylation in all CpG-density intervals (Figure 4E). Together, our results indicate that the methylation level of the area is more important than CpG number in determining AMD.

### Age-associated transcriptional drift reduces the gene-to-gene disparity of transcriptional activity

The steady expansion of an epigenomic disorder impairs transcriptome homeostasis and increases transcriptional noise. The SiNG-PCRseq approach, which reliably measures transcript levels, particularly of low-set expression level genes [29], was used to investigate the transcriptional activity of genes in the aged spleen. In addition to the spleen samples used for methylation analysis, we included additional spleen samples of ages 2m (n=2), 6m (n=3), and 12m (n=2) for gene expression analysis. We also used a reference sample, consisting of a mixture of mouse and rat genomic DNAs, to correct amplification biases. We used a set of primer pairs that can amplify 424 target exonic sequences from 257 genes of both mouse and rat, including 175 genes identified as epi-drivers [35]. Rat genomic DNA was utilized as a spike-in in multiplexed SiNG-PCRseq to accurately quantify mouse cDNA sequences compared to matched rat gDNA sequences. To ensure equal amplification and to establish detectable flags between the cDNA-derived reads and the rat genomic DNA reads after sequencing, each primer pair was designed to contain one or more nucleotide differences between the amplicons of different species. After SiNG-PCRseq, we acquired 2.2×10^8^ reads from triplicate libraries of 20 samples (3.7×10^6^ ±0.6×10^6^ reads/library), with around 76% of them mapping perfectly to our amplicon reference. The M/R ratio of each exonic target, comparing mouse read counts to rat counts, underwent quality filtering for low read numbers and extensive amplification biases, resulting in a set of 288 exonic targets from 206 genes. After filtering, the data were processed for amplification bias correction and normalization, allowing us to evaluate the expression level of each exonic sequence across all samples (Supplementary File 1). For instance, among DNA methyltransferases (Dnmt) genes, *Dnmt1* was less expressed with age whereas *Dnmt3b* and *Dnmt3l* were more expressed at 23 months (Supplementary Figure 6).

**Figure 5:**
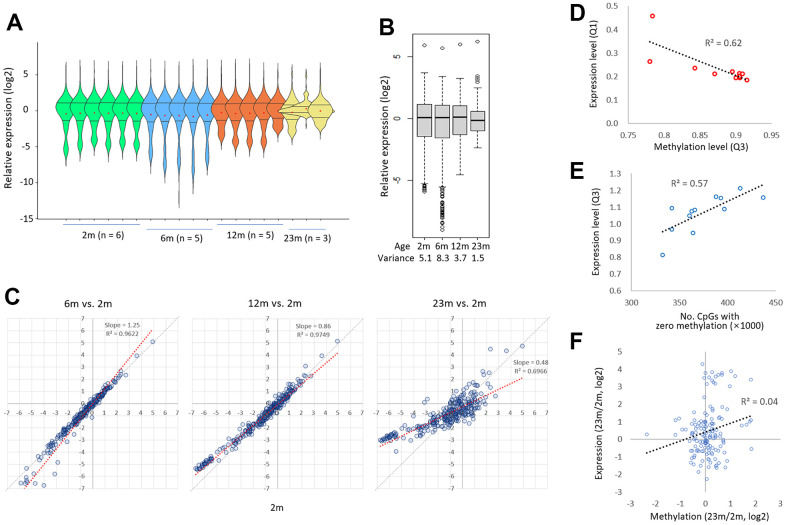
**Age-related diminution in the gene-to-gene disparity of transcriptional activity.** (**A**) A comparison of the expression levels of 285 exonic sequences from 175 epi-driver genes in 2m, 6m, 12m, and 23m individual samples. The Q1 and Q3 quartile-level lines along with the mean levels are shown (dots in red). (**B**) A comparison of expression levels of 285 exonic sequences by age group. Variances are shown below. The expression level was assessed by comparing the PCR amplicon counts of mouse cDNA and rat gDNA (M/R ratio; see text for details). (**C**) Patterns of transcriptional drift for genes with low and high transcriptional activity. The expression levels of target genes in 2m samples were compared to those in 6m, 12, or 23m samples. The thick black arrows denote the direction of change in expression levels in low-expressing genes (left, ‘hyper-transcription’) and high-expressing genes (right, ‘hypo-transcription’) with age. Grey and blue lines represent the reference and trend lines, respectively. (**D**) Correlation between methylation loss and gene expression increase. The third quartile (Q3) methylation level, which decreases upon hypomethylation, was plotted against the first quartile (Q1) expression level in the same sample. The regression curve shows a significant negative correlation between these two variables (*R^2^*=0.72). (**E**) Correlation between methylation gain and gene expression decrease. The number of zero methylation sites, which decreases upon hypermethylation, was plotted against the Q3 expression level in the same sample. The regression curve shows a significant positive correlation between these two variables (*R^2^*=0.43). (**F**) No relationship between methylation change and expression change (23m/2m) in the epi-driver genes.

The violin plots revealed that the distribution of expression levels among the genes was the broadest in the 6m group, then shrunk significantly in the 12m group, and finally narrowed in the 23m group (Figure 5A). As a result, the 6m group had the largest variance in the mean gene expression levels, but it was decreased to less than one-fifth in the 23m group (Figure 5B). Furthermore, as shown by the lowered slope of scatter plot in Figure 5C, those genes that were weakly expressed in young spleen became more transcribed in old, whereas those genes which were relatively well expressed in young tended to diminished in old. We also observed a strong correlation between the third quartile values of CpG methylation levels and the first quartile values of gene expression levels across all samples analyzed (*R^2^* = 0.62; Figure 5D, left). This suggests a potential link between the global loss of methylation in highly methylated regions and the increase in transcription of lowly expressed genes. We also found that the number of CpG sites with no methylation decreased with age and was moderately correlated with the first quartile values of gene expression (*R^2^* = 0.57; Figure 5E). This implies another link between methylation gain in lowly methylated regions and the decrease of expression levels. We previously identified a similar age-associated bidirectional change in transcriptional activity in mouse CD4-positive T cells [36]. Together, our results suggest that age-associated bidirectional transcriptional drift occurs, resulting in a reduction in disparities in expression levels between strongly and weakly expressed genes. This age-associated transcriptional drift pattern mirrors the AMD pattern, in that genes with a lower-level transcript experience ‘hyper-transcription’ whereas those with a high-level transcript show ‘hypo-transcription’. Meanwhile, there was no obvious association (*R*^2^ = 0.04) between methylation alteration and expression change (23m/2m) in the epi-driver genes (Figure 5F, see Discussion).

## DISCUSSION

The mean methylation levels of all CpG sites available in RRBS decreased from 41% to 38% as age advanced. The overall decrease in DNA methylation, however, does not necessarily imply a decrease in DNA methylation at every CpG site. Individual CpG methylation levels do fluctuate, but these fluctuations are predictable: rising for CpGs with low methylation levels or lowering for CpGs with high methylation levels, both moving towards the mean level. The change is not restricted to singleton CpGs but also extends to genomic areas with clustered CpGs, and it is influenced by the CpG density and methylation level of the region, both of which are inversely correlated: hypermethylation in promoters with low methylation levels and dense CpGs, and hypomethylation in gene-body and intergenic regions with high methylation levels and sparse CpGs. This age-associated, bidirectionally occurring methylation drift exhibits the “regression toward the mean” tendency, indicating that the global methylation shift during aging is driven by two opposing, antithetical forces. Recent methyl-binding protein capture and sequencing (MBD-seq) of aged skeletal muscle DNA in mice revealed a regression-to-the-mean pattern of methylation drift [37]. Unlike our RRBS methylome, this study includes DNA methylation data on various genomic repeats and demonstrates that a variety of genomic transposable elements (TE) such as endogenous retroviruses (ERVs), long and short interspersed elements (LINE1s and SINEs, respectively) undergo hypermethylation in lowly methylated repeated copies and hypomethylation in highly methylated repeated copies as a function of age. According to current and previous studies, this “regression toward the mean” trend occurs globally and, as a result, diminishes the region-to-region variance in the genomic landscape of DNA methylation in aged tissues.

This regression-toward-the-mean pattern of epigenetic drift could be the result of a relaxation of epigenetic control over the genome caused by chaotic cellular conditions that increase with age. During adolescence, methylation states are closely maintained at a high cost of cellular energy to ensure chromatin stability and transcriptional equilibrium of adjacent genes. However, as energy resources dwindle and age-associated dysregulation accumulates across the chromosomes, the genome gradually releases itself from the high-energy tension required to maintain the extremely sophisticated epigenomic architecture and thus fails to keep the status quo, displaying a genome-wide regression-toward-the-mean drift of DNA methylation. As a result, preexisting methylation disparities between genomic regions formed during ontogenesis may gradually diminish with age [37].

An important topic concerns the functional repercussions of regression-to-the-mean epigenetic drift. A few papers reported that the link between age-driven DNA methylation and gene expression changes is, at best, tenuous [20, 38–40]. In this study, we did not investigate specific gene promoters or loci implicated in the regulation and maintenance of splenic function. Our result may therefore not be applicable when discussing the specific effect of the methylation drift and transcriptional drift on the function and physiology of spleen. Nevertheless, we can envision that, as a result of global on-going hypo- and hypermethylation events, a large number of genes in the spleen tissue, either transcriptionally permissible or not, may face continuously changing circumstances; gene promoters may be gradually more accessible to transcriptional activators and repressors, resulting in a regression-to-the-mean transcriptional drift across the genes. Related genes with increasing expression variability in old tissue may be drivers to phenotypic diversity, elevating the heterogeneity within cell populations [41, 42], and likely contribute to disease vulnerability, diversion to and exacerbation of aging symptoms, and alterations in gene networks in the spleen tissue. Notably, we previously showed that the transcriptional drift was not observed in muscle or brain tissues whereas splenic T cells isolated from the same old mice clearly proved the occurrence [36]. Moreover, the shift was amplified in the splenic T cells derived from the Huntington model mice (YAC128) that exhibit symptoms of accelerated aging [36]. According to the findings, the transcriptional drift starts early or late, depending on the tissues/organs, and increases with age. Similarly, the splenic epigenetic/transcriptional drift may broaden its effect with age. However, the stochastic nature of the changes leads to unpredictable differences in the methylome/transcriptome among aging individuals [43] and makes it difficult to forecast which genes tend to be affected and what ramifications it may have in the spleen.

To precisely delineate the expression changes driven by epigenetic drift, we have employed SiNG-PCRseq in our study. While RNAseq approaches have limitations in detecting expression changes for lowly expressed genes due to their dependence on read depth, SiNG-PCRseq overcomes this by utilizing competitive amplification of targets and competitors. This enables quantitative analysis of all target genes with robust enrichment and high sensitivity across expression levels. Our study focuses on uncovering subtle expression changes during aging, specifically in genes with low expression profiles. Despite the limited number of genes analyzed, we have chosen this targeted approach as it aligns better with our study objectives. From the result of SiNG-PCRseq, we observed such a bimodal transcriptional activity change, with the upregulation of low expression level genes and the downregulation of high expression level genes. However, as in the previous studies above [20, 38–40], we failed to detect a link between age-associated DNA methylation changes and gene expression changes. Given that multiple layers of the epigenetic system are involved in gene transcriptional control, it is not surprising that a change in DNA methylation alone is insufficient to affect transcription activity [22, 23, 44, 45]. If this is the case, the bimodal transcriptional drift could be understood as the outcome of a regression-toward-the-mean movement orchestrated by numerous epigenetic variables, including histone modifications. In aged human and rodent tissues and cells, for instance, reduced levels of histone H3-lysine 9 (H3K9) trimethylation, which is required for maintaining CpG methylation level in heterochromatin [46], have been found [47–49].

Finally, failure to maintain epigenomic and transcriptomic homeostasis with age may lead to a global dysregulation of cell and tissue function. We were curious about how well and consistently the quantitative epigenome organization formed in young life is preserved in aged spleen. We demonstrated in this study that age-associated methylation drift is primarily determined by the region’s methylation level and CpG density milieu, each as a primary and secondary contributing factor, and follows the regression-toward-the-mean tendency, resulting in a decrease in methylation-level disparity between highly and weakly methylated genomic regions. We anticipate that our findings in this work will open the way for future investigation into the significance of epigenetic drift and its effects on cellular physiology, as well as establishing a causal role of epigenetic mechanism in aging.

## MATERIALS AND METHODS

### Mouse and nucleic acid preparation

Genomic DNAs and RNAs were simultaneously extracted from the spleen tissues using All Prep® DNA/RNA Mini Kit (Qiagen, Germany) following the manufacturer’s instruction.

### Reduced representation bisulfite sequencing (RRBS)

Bisulfite-converted MspI-restricted genomic fragments were molecularly indexed and sequenced using an in-house developed RRBS method (Supplementary Figure 1). In this method, we devised two different adaptors with a CpG overhang in one end. Adaptor-A carries a molecular barcode and the binding site for Illumina R1 sequencing primer. Adaptor-B has the binding site for Illumina R2 sequencing primer. All the cytosines on the Illumina primer binding sites of two adaptors were protected from bisulfite conversion as they were priorly methylated during synthesis. Fragmented genomic DNAs (100ng) were purified using Zymo DNA clean-up Kit (Zymo Research, USA) and ligated with the adaptors (2 pmol/each) at 25° C for 30 min using Blunt/TA Ligase Master Mix (NEB, USA). The recessive part of the ligates was then filled in using Tag DNA polymerase (Solgent, Korea) at 72° C for 30 min, which concomitantly removes the unligated adaptor strand. In this Fill-in step, dCTP was replaced with methyl-dCTP. Bisulfite conversion was performed for the adaptor-attached MspI fragments using the EZ DNA Methylation-Gold™ Kit (Zymo Research, USA) following the manufacturer’s instructions. After bisulfite conversion, MspI fragments were subsequently amplified by two successive rounds of PCR to generate sequencing-ready libraries. First PCR was performed using primers, P1 and P2, for 10 cycles of 95° C for 30sec, 60° C for 30sec, and 72° C for 30 sec. Second PCR was performed using index primers, idxP1, and idxP2, for 10 cycles of 95° C for 30 sec, 60° C for 30 sec, and 72° C for 30 sec. Massive parallel sequencing was performed on Illumina Hiseq platform with 151 bp paired-end reading.

### SiNG-PCRseq

Total RNA (1ug) was converted to cDNA using iScript cDNA Synthesis Kits (Bio-Rad, USA). SiNG-PCRseq was performed using the cDNA and the rat genomic DNA as a competitor array for the target genes as described previously. Briefly, we composed 10 primer pools using 424 primer pairs and used each in multiplexed PCR with 15 ng of mouse cDNA and 2 ng of rat gDNA as templates in the following conditions: 95° C for 15 min, 45 cycles of 95° C/20 sec, 57° C /40 sec, 72° C/1 min, followed by a final extension at 72° C for 5 min. The Amplicons were pooled together for each sample and purified using Expin purification kit (GeneAll, Korea), 5’-end phosphorylated using T4 polynucleotide kinase (NEB) by incubating at 37 for 30 min, ligated with NGS adapters using T4 ligase (NEB) at 25 for 2 hours and amplified using idxP1 and idxP2 to generate sequencing libraries with following conditions: after 98° C for 15 min, 20 cycles of 98° C/20 sec, 68° C/30 sec, and 72° C/1 min, followed by a final extension at 72° C for 5 min. Three independent libraries were prepared for each sample to ensure reproducibility. Additionally, a library of genome mixture of mouse and rat was generated in triplicate and used to correct for any amplification biases in the target DNA. Massive parallel sequencing was performed on Illumina Nextseq platform with 151 bp single-end reading.

### Sequence analysis

The read data from the RRBS sequencing were processed using trim_galore (version 0.6.6) to remove the adaptors. The cleaned reads were then aligned to the mouse reference genome (mm10) using the Bismark package (version 0.22.3). The methylation status of each CpG site was determined using ad hoc Perl scripts. Clonal copies were identified by the presence of the same molecular barcode and were removed by deduplication. The methylation levels of CpG sites were collected, and only those with at least 10 deduplicated reads in each sample were selected for further analysis. To compare the global and regional methylation patterns between samples, only CpG sites with qualified methylation levels from at least 13 samples were used in the analysis. The promoter region was defined as the region of 2 kilobases surrounding the transcription start site (TSS ± 1 kilobase), the gene body region was defined as the region from the start to end described in the annotation files, and the intergenic region was defined as any other region. To capture the collective influence of neighboring CpG sites, the intergenic CpG block was operationally defined as the region where any two neighboring CpG sites are less than 1 kilobase apart from each other in the intergenic region.

The read data from SiNG-PCRseq were analyzed essentially as described. Briefly, adaptor-trimmed sequences were identified for their target and species origin by aligning them to the target DNA sequences using the BLAST program. The count data for each target sequence from each sample were obtained and subjected to a quality filter. Targets were excluded if any reference library had fewer than 100 read counts or if any sample library had read 300 counts. The count data for each target amplicon from each sample were utilized to determine the mouse fraction. Targets with extensive amplification bias in the reference sample (<0.3 or >0.7) were excluded from the analysis. The amplification biases present in the mouse fractions of the remaining targets were corrected by employing the mouse fractions of the reference sample as previously described. The bias-corrected fractions were then converted to relative quantities of the rat amplicon and normalized by using the average relative quantities of the library. The final expression values of a sample were determined by averaging the relative quantities obtained from the triplicate libraries of the sample.

### Data availability

Sequencing data were deposited under GSE203289.

## Supplementary Material

Supplementary Figures

Supplementary File 1
