# Supplementary material for: Parallel shift of DNA methylation and gene expression toward the mean in mouse spleen with aging
Source: Aging (Albany NY). 2023 Jul 25;15(14):6690–709. doi: 10.18632/aging.204903 (PMC10415566; doi:10.18632/aging.204903)
Supplement: Supplementary Figures [file aging-15-204903-s001.pdf]

SUPPLEMENTARY FIGURES

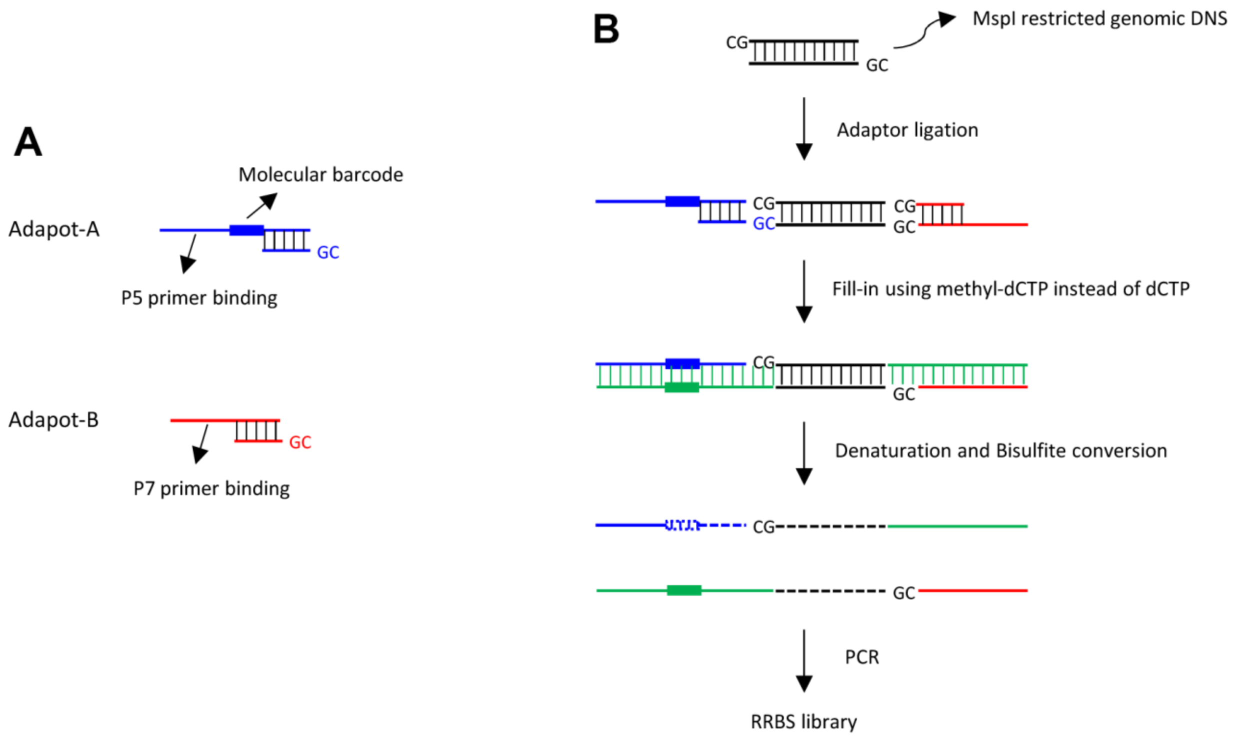

Supplementary Figure 1. Scheme for RRBS library construction. (**A**) Our RRBS method utilizes two adaptors, depicted schematically, both of which have a partially double-stranded DNA structure with a CG overhang in their duplex region. Adaptor-A and Adaptor-B both contain an Illumina sequencing primer binding site (R1 and R2, respectively). Additionally, Adaptor-A has an 8-nucleotide random barcode between the primer binding site and the duplex region. The cytosine residues in the primer binding sites are pre-methylated to prevent their conversion by bisulfite. The 5' end of the strand with the CG overhang lacks a phosphate group, making it unable to participate in the ligation reaction. (**B**) The RRBS process is depicted in a schematic representation. After restriction by MspI, the DNAs are ligated with adaptors A and B, as shown in the figure. Only one form of ligation is depicted where both adaptors are attached to an MspI fragment, however, other forms of ligation are also possible, where the same adaptors are attached to both ends of a DNA fragment. These types of ligation can result in PCR suppression due to intra-strand duplex formation between the adaptor sequences. Following ligation, the fill-in reaction using dNTPs takes place, where dCTP is substituted with methyl-dCTP to make the nascent DNA resistant to bisulfite conversion. The double-stranded DNAs are then denatured and subjected to bisulfite conversion. Finally, the bisulfite-converted DNAs are amplified using primers for the Illumina sequencing platform and a sample index, generating the sequencing-ready library.

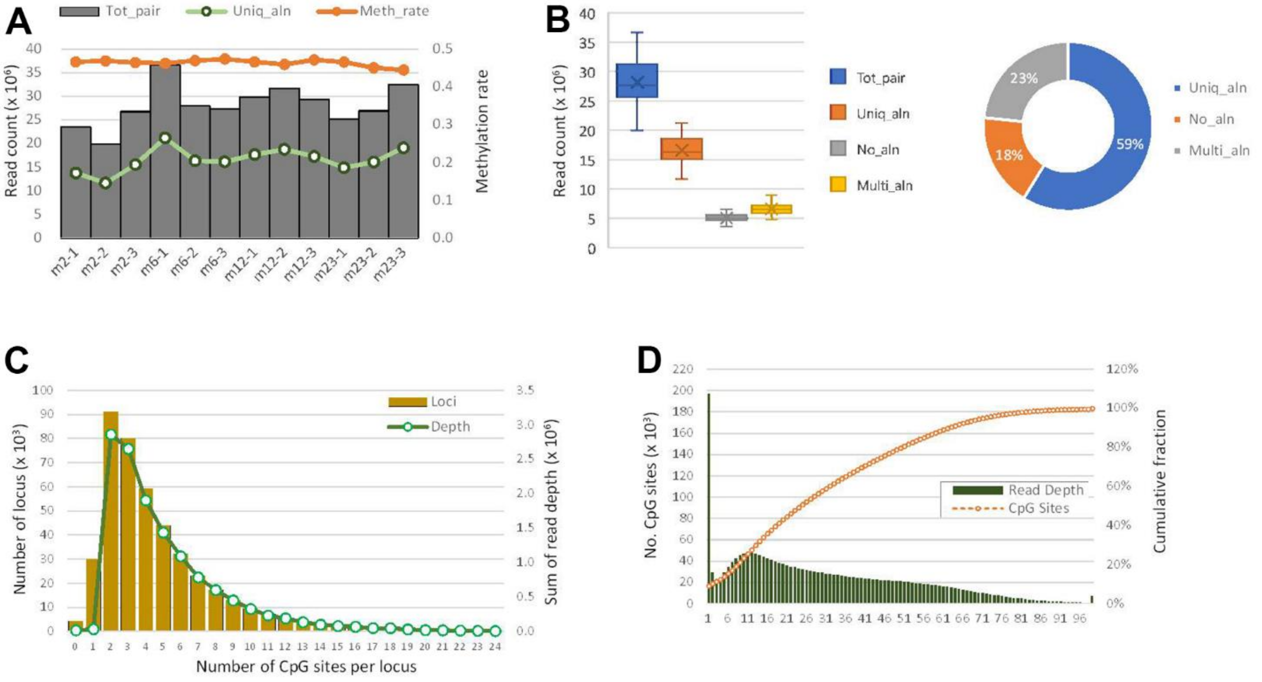

Supplementary Figure 2. RRBS data information. (**A**) The distribution of unique alignment sequences (Uniq_aln) across each sample is shown, along with the total number of sequencing reads (Tot_pair) and the fraction of methylated CpGs out of the total CpGs (Meth_rate) (**B**) The read profile following Bismark alignment is depicted in a box plot for total reads, uniquely aligned reads (Uniq_aln), reads aligned to multiple loci (Multi_aln), and unaligned reads (No_aln) on the left. The proportion of reads for each alignment pattern is illustrated in a pie chart on the right. (**C**) All mapped genomic loci were classified based on the number of CpG sites present in their sequences. The frequency of each category is displayed in a bar chart on the left axis, with the total number of deduplicated reads mapped on each category on the right axis. (**D**) The abundance of CpG sites with the given number of deduplicated read depths is plotted along with the accumulated fraction of CpG sites from low depth to high depth.

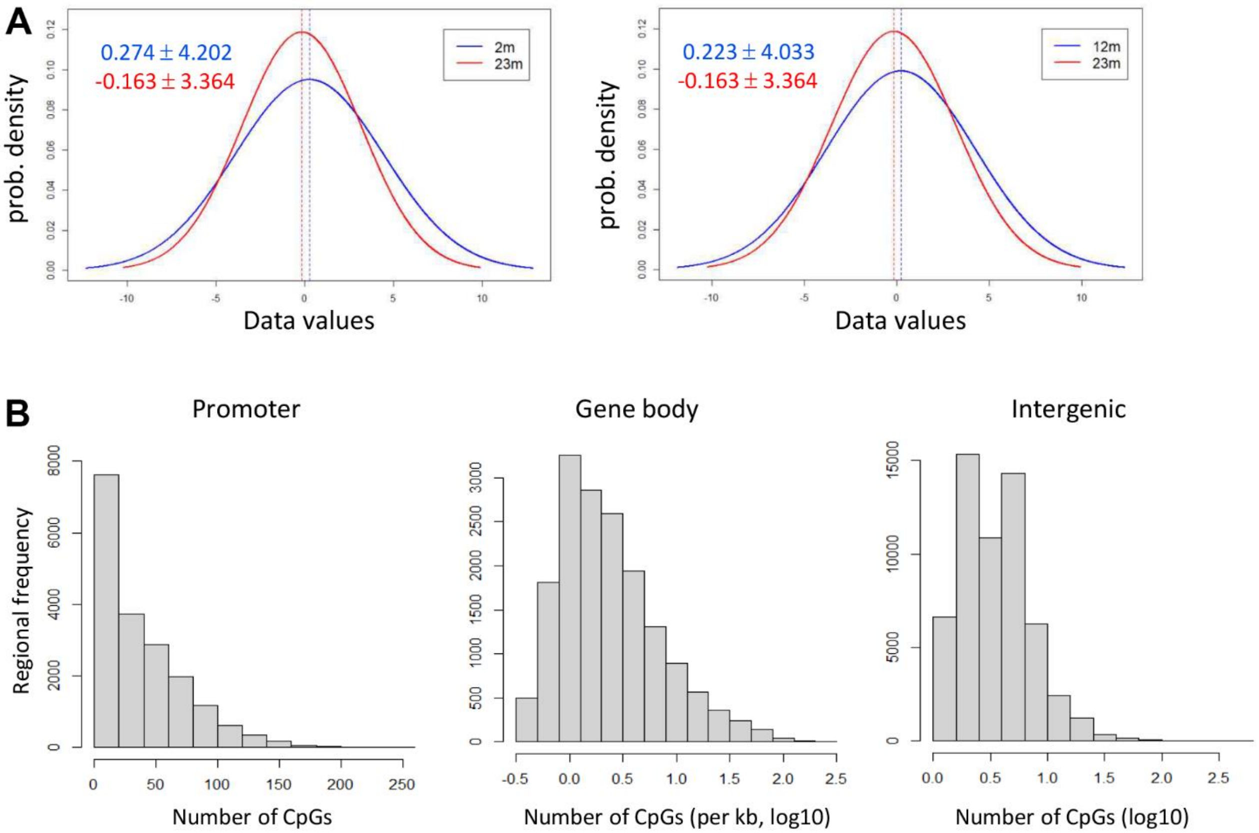

Supplementary Figure 3. (**A**) The normal distribution patterns of methylation levels (m-values) in 2m or 12m samples (blue) and 23m (red) samples. The sample means are represented by vertical lines. The sample means and the standard deviations are shown (blue for 2m or 12m and red for 23m). (**B**) The frequency of genomic regions across intervals of CpG numbers for different genomic structures/functions (promoters, gene bodies, and intergenic regions).

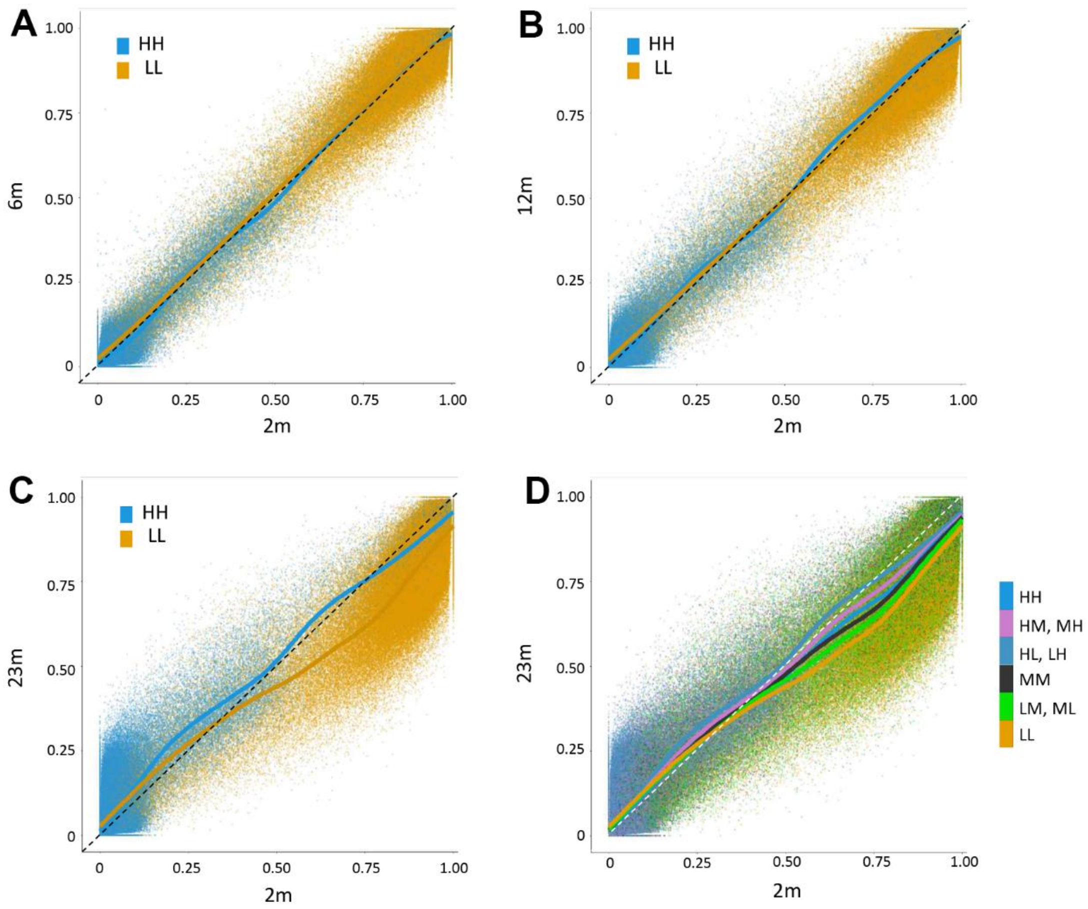

Supplementary Figure 4. Scatter plots of 6m (**A**), 12m (**B**), 23m (**C**), and combined samples (**D**) for methylation levels of HH (blue) and LL (yellow) labeled CpGs versus 2m samples. Each dot corresponds to a single CpG site. Dashed lines indicate the 1:1 reference line and thick lines denote the regression lines of given classifications.

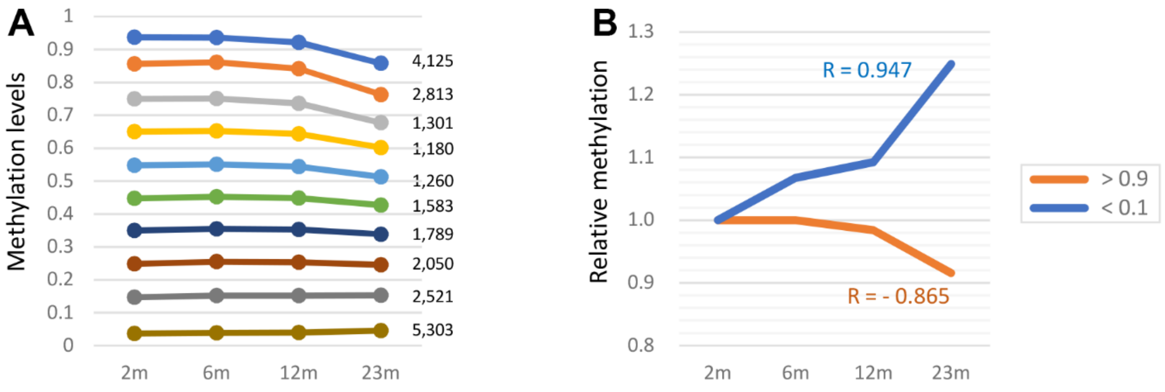

Supplementary Figure 5. Dependence of the age-linked methylation-change pattern on the region's methylation level. In (**A**) gene-body regions were grouped by methylation level (0.1 intervals of b-value, y-axis) as indicated by lines in different colors. The number of genebody regions in each methylation interval is shown. In (**B**) a contrasting pattern of age-associated methylation change is shown between the low-methylation (<10%, orange) and high-methylation (>90%, blue) groups. Pearson correlation (R) coefficient is indicated.

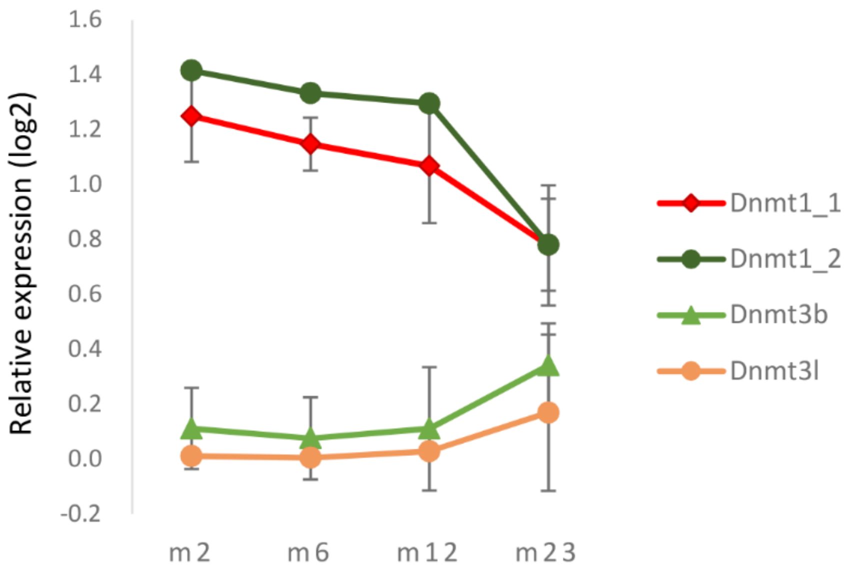

Supplementary Figure 6. Expression level of DNA methyltransferase (Dnmt) genes. From the SiNG-PCRseq, the expression levels of *Dnmt1, Dnmt3b*, and *Dnmt3l* were determined by M/R ratio (see text for details). Two different *Dnmt1* exonic sequences as indicated by Dnmt1_1 and Dnmt1_2 were chosen for analysis. Error bars indicate the standard deviation.
